# Exosomal MicroRNAs: An Emerging Important Regulator in Acute Lung Injury

**DOI:** 10.1021/acsomega.3c04955

**Published:** 2023-09-22

**Authors:** Bowen Lan, Xuanchi Dong, Qi Yang, Yalan Luo, Haiyun Wen, Zhe Chen, Hailong Chen

**Affiliations:** † Department of General Surgery, The First Affiliated Hospital of Dalian Medical University, Dalian 116000, China; ‡ Laboratory of Integrative Medicine, The First Affiliated Hospital of Dalian Medical University, Dalian 116000, China; § Department of Traditional Chinese Medicine, The Second Affiliated Hospital of Dalian Medical University, Dalian 116023, China; ∥ Institute (College) of Integrative Medicine, Dalian Medical University, Dalian 116044, China

## Abstract

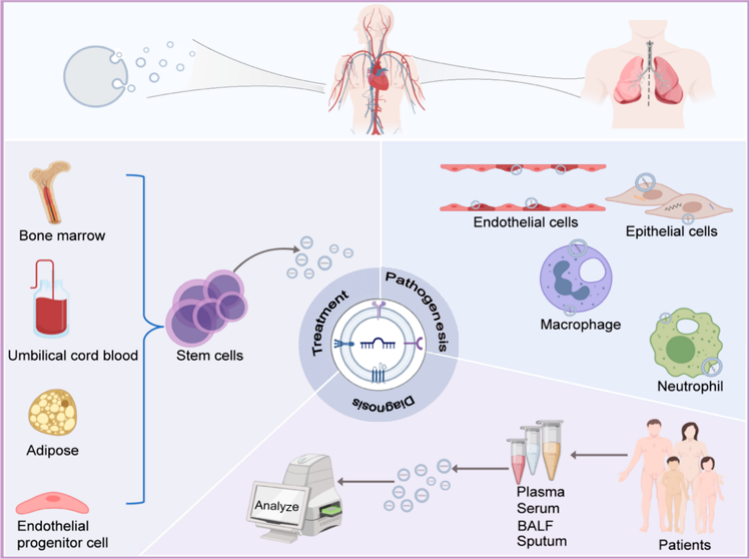

Acute lung injury (ALI) is a clinically life-threatening form of respiratory failure with a mortality of 30%–40%. Acute respiratory distress syndrome is the aggravated form of ALI. Exosomes are extracellular lipid vesicles ubiquitous in human biofluids with a diameter of 30–150 nm. They can serve as carriers to convey their internal cargo, particularly microRNA (miRNA), to the target cells involved in cellular communication. In disease states, the quantities of exosomes and the cargo generated by cells are altered. These exosomes subsequently function as autocrine or paracrine signals to nearby or distant cells, regulating various pathogenic processes. Moreover, exosomal miRNAs from multiple stem cells can provide therapeutic value for ALI by regulating different signaling pathways. In addition, changes in exosomal miRNAs of biofluids can serve as biomarkers for the early diagnosis of ALI. This study aimed to review the role of exosomal miRNAs produced by different sources participating in various pathological processes of ALI and explore their potential significance in the treatment and diagnosis.

## 1 Introduction

Acute lung injury (ALI) is one of the most common critical illnesses of the respiratory system. It is an acute hypoxic respiratory insufficiency caused by various direct or indirect causes other than of cardiac origin. Its worsened form is acute respiratory distress syndrome (ARDS).^1,2^ ARDS was first widely recognized in 1994. The American–European Consensus Conference proposed to diagnose ARDS using four criteria: acute hypoxic episode, bilateral pulmonary infiltrates, pulmonary artery wedge pressure ≤18 mmHg or the absence of clinical manifestations of left atrial hypertension, and a level of the arterial pressure of oxygen/inspiratory fraction of oxygen (PaO_2_/FiO_2_) ≤ 200 mmHg.^3^ ALI was identified using diagnostic criteria similar to those for ARDS except for PaO_2_/FiO_2_ ≤ 300 mmHg. In 2012, the Berlin definition revised and supplemented the diagnostic standard for ARDS.^4^ According to PaO_2_/FiO_2_, ARDS was classified as mild (PaO_2_/FiO_2_: 200–300 mmHg), moderate (PaO_2_/FiO_2_: 100–200 mmHg), or severe (PaO_2_/FiO_2_ < 100 mmHg). In the United States, there are approximately 200,000 patients with ALI and 75,000 deaths due to ALI per year.^5^ Nationwide, there are approximately 3 million cases of ALI per year, which account for 10% of all patients in intensive care units,^6^ and the mortality rate is 35%–40%.^7^ ALI is caused by pneumonia, severe infections, trauma, shock, burns, acute pancreatitis, radiation injury, blood transfusion, and other conditions.^8^ Hyaline membrane formation and widespread pulmonary edema are the pathologic characteristics.^9−11^ Some patients with severe ALI develop ARDS, which may be accompanied by irreversible pulmonary fibrosis, resulting in pulmonary dysfunction.^12^ Currently, a new understanding of the pathogenesis of ALI has emerged. The significant imbalance of inflammatory responses in the lungs and whole body is the key theory of the pathogenesis of ALI. The disturbance of the coagulation system, dysregulation of vasoactive chemicals, defective regulation of the alveolar–capillary barrier, and imbalance of oxidative stress and apoptosis are additional pathogenic mechanisms implicated in the regulation.^13^ Despite decades of research, the primary treatment for ALI is still symptomatic and supportive, and there is no particularly efficient treatment in clinical practice. Drug therapy mainly aims to improve lung inflammation and oxygenation. Commonly used drugs include corticosteroids and inhaled vasodilators.^14^ Mechanical ventilation is thought to be the only supportive therapy that can enhance the survival rate of patients with ALI.^15^ However, prolonged mechanical ventilation can result in ventilator-associated lung injury.^16,17^ To improve a patient’s prognosis and reduce mortality, it is essential to investigate treatments for ALI and markers that can assist in its early diagnosis.

Exosomes are extracellular vesicles (EVs) with diameters from 30 to 150 nm.^18^ They were thought to be a cellular waste product that only discharged intracellular and membrane components from the cell when first detected during sheep reticulocyte development into mature erythrocytes.^19,20^ However, it is now considered an emerging intercellular communication vehicle, and their various cargoes (proteins, lipids, lncRNA, miRNA, and mRNA) play a crucial role in the pathophysiological mechanisms,^21−23^ treatment,^24−26^ and even diagnosis^27,28^ of diseases. Since the discovery of miRNAs more than 2 decades ago, researchers have developed novel perspectives of diseases, making miRNAs promising therapeutic targets.^29^ Multiple types of pulmonary cells cooperate in controlling lung inflammation during ALI.^30^ Exosomes transmit a diversity of specific miRNAs across cells stably and perform various pathogenic regulatory functions in this process.

Therefore, this paper summarizes the current research on the pathogenesis and treatment of ALI based on the participation of exosomal miRNAs in cellular interactions and describes its potential applications in diagnosis.

## 2 Exosomes

EVs are important mediators of intercellular communication and play important roles in physiological and pathological processes. They can be divided into exosomes, microvesicles, and apoptotic bodies. The exosome is a type of vesicle secreted by various cells and has a bilayer membrane structure.^18,31,32^ The International Society for EV (ISEV) has published the latest definition of different subtypes of EVs in “Minimum Information for Studies of Extracellular Vesicles 2018” (MISEV 2018).^33^ This section discusses the formation, morphology, sources, molecular composition, and functions of exosomes.

### 2.1 Formation

**Figure 1:**
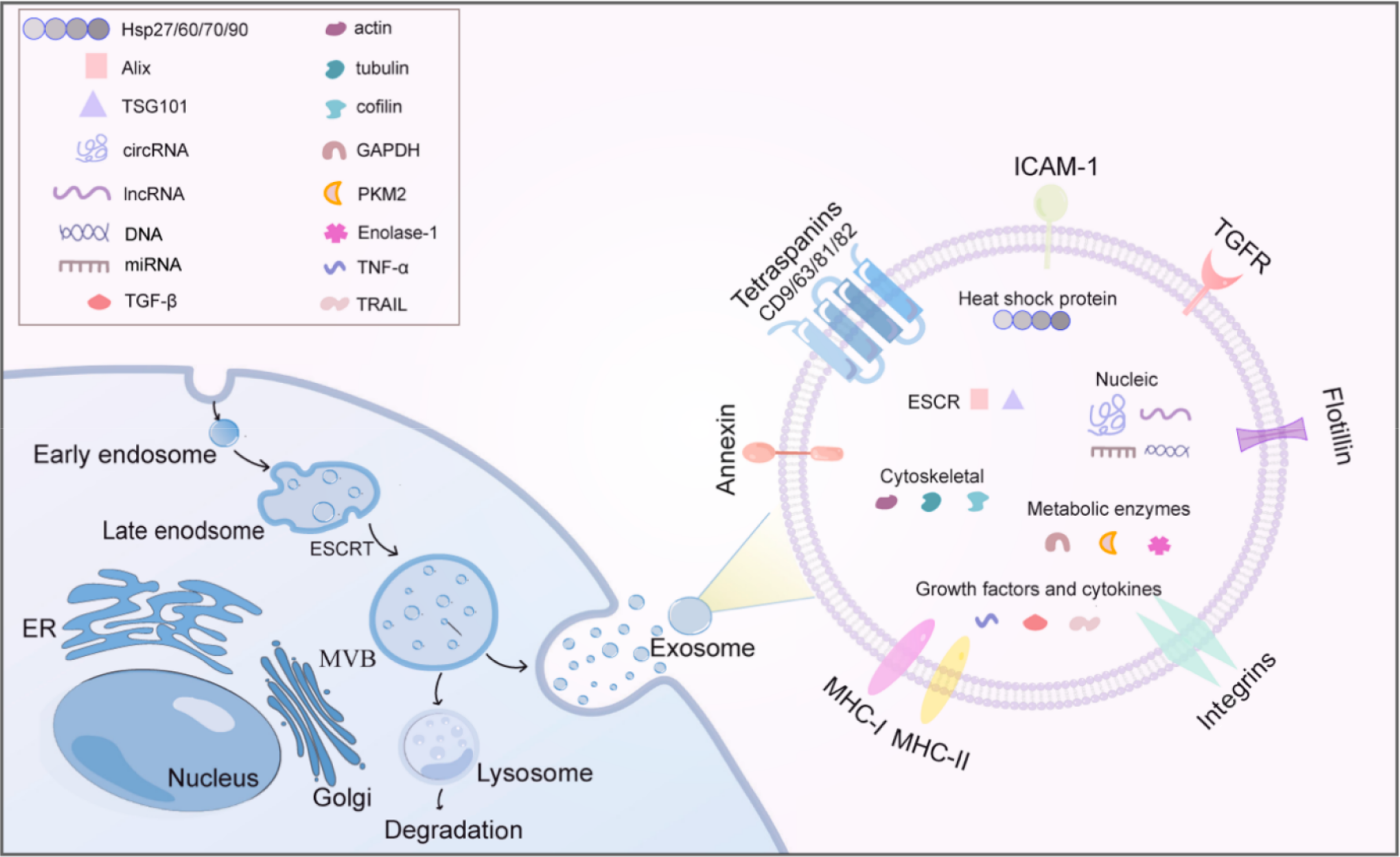
Biogenesis and molecular composition of exosomes. (Left) Schematic representation of the exosome release into the extracellular space. The generation of exosomes begins with endocytosis of the cell membrane and undergoes several steps within the cell before finally fusing with the cell membrane to be transported to the extracellular space by exocytosis. (Right) Schematic diagram of the major components of exosomes. All exosomes have a typical structure similar to that of a cell, including proteins (tetraspanins, annexin, heat shock proteins, etc.), lipids (ceramide, cholesterol, phosphatidylserine, sphingolipid, etc.), and genetic material (DNA, mRNA, miRNA, circRNA, lncRNA, etc.).

In 1983, exosomes were first discovered by Pan and Johnstone during the maturation of sheep reticulocytes and were associated with the release of transferrin receptors from sheep reticulocytes into extracellular space.^19,34^ In 1989, these functional EVs were formally defined by Johnstone as exosomes.^35^ The biosynthetic pathways of exosomes include the endosomal and plasma membrane pathways, among which the endosomal pathway is widely recognized.^36^ In the endosomal pathway, they are initially produced as early endosomes produced by the inward budding of cell membranes. Subsequently, intracellular bioactive substances accumulate in the early endosome, forming the late endosome. The late endosome membrane buds inward to generate many small vesicles within the cell, eventually coalescing into multivesicular bodies (MVBs) via the Golgi apparatus. Lysosomes within the cell degrade some MVBs, and the others are fused with the cell membrane and release small vesicles to the extracellular space through exocytosis. These are called exosomes (Figure 1).^37^ MVB synthesis is the central process of exosome biogenesis, mainly through endosomal sorting complexes required for a transport (ESCRT)-dependent pathway.^38^ However, several laboratories have found that exosome biogenesis is not substantially reduced after the ESCRT pathway is inhibited.^39^ Other generation pathways reported so far have been classified as ESCRT-independent mechanisms. Studies have shown that the Rab protein could regulate the occurrence of exosomes through endosomes and plasma membranes, among which Rab27a and Rab27b could participate in the localization of vesicles. Rab27a could dynamically regulate plasma membrane phosphatidylinositol 4,5-bisphosphate (PIP2) to assemble plasma membrane microdomains and participate in membrane germination, while Rab35 could regulate PIP2 levels in cell membranes. Rab11 was involved in exosome formation through calcium-induced MVB fusion.^40^ In addition, cortactin, Rab27a, and coronin 1b collaboratively modulate cutaneous actin to promote exosome secretion.^41^

### 2.2 Morphology

With the application of a scanning electron microscope in transmission mode, transmission electron microscopy (TEM) is widely used for the morphological characterization of exosomes with an imaging resolution of approximately 1 nm. Exosomes are negatively stained and cup-shaped under TEM.^42^ However, TEM must be operated under vacuum conditions, and the exosome samples must be dyed, fixed, and dehydrated. These procedures impact the actual morphology and size of exosomes. Recently, it has been reported that the morphological characterization of exosomes using cryo-electron microscopy is more representative of the natural morphology of exosomes because there is no need to perform the above procedures on the sample. Under cryo-electron microscopy, exosomes are mostly spherical.^43^ This provides fresh perspectives on the morphological characteristics of exosomes. Due to the heterogeneity of exosomes, the morphology of exosomes may also be diverse. Hoog et al. found that the morphology of EVs was varied by cryo-electron microscopy, which may be used to distinguish different exosome subgroups.^44^

### 2.3 Sources

Almost all living cells can secrete exosomes, especially dendritic cells (DCs), epithelial cells, endothelial cells, and lymphocytes.^45^ Therefore, exosomes can be extracted from a wide range of biofluids (serum, plasma, alveolar lavage, saliva, urine, peritoneal lavage, and breast milk)^46−51^ and cell supernatants (stem, immune, and tumor cells).^52−55^ Recent reports have shown that various edible plants can also produce exosomes, such as grapes,^56^ apples,^57^ ginger,^58^ citrus lemon,^59^ and broccoli.^59^ These findings have certainly enriched our understanding of the origin of exosomes.

### 2.4 Molecular Composition

The molecular composition of exosomes mainly consists of proteins, nucleic acids, lipids, and other immunomodulatory factors. The proteins can be divided into two major groups: the membrane and intramembrane. The membrane proteins mainly include tetraspanins (CD9, CD63, CD81, and CD82), flotillin, annexin, antigen-presenting molecules (major histocompatibility complex I [MHC I] and major histocompatibility complex II [MHC II]), and adhesion molecules. Intramembrane proteins mainly contain heat shock protein (Hsp) family proteins (Hsp27, Hsp60, Hsp90, and Hsp70), ESCRT proteins (ALG-2-interacting protein X [Alxi] and tumor susceptibility gene 101 [TSG101]), cytoskeletal proteins (actin, tubulin, and cofilin), growth factors and cytokines (tumor growth factor-beta [TGF-β], tumor necrosis factor-alpha [TNF-α], and TNF-related apoptosis-inducing ligand [TRAIL]), metabolic enzymes (glyceraldehyde 3-phosphate dehydrogenase [GAPDH], enolase-1, and pyruvate kinase M2 [PKM2]), signal transduction factors (melanoma-associated molecules, ADP ribosylation factor 6 [ARF6], and cell division cycle 42), and adhesion molecules (milk fat globule-EGF factor 8, integrins, and P-selectin). Lipids mainly contain ceramide, cholesterol, sphingolipid, and phosphatidylserine. Nucleic acids mainly include DNA and RNA. RNA has mRNA and noncoding RNA, of which noncoding RNA is dominated by miRNA. Among various exosome molecular components, CD9, CD81, TSG101, CD63, and flotillin are currently considered biomarkers of exosomes because of their stable expression within exosomes.^60,61^

### 2.5 Functions

Exosomes are highly stable and immunogenic with low toxicity, which has sparked great interest in them as intercellular communicators. There are three modes of exosome communication action: internalization by the recipient cell, delivering cargo such as proteins, and nucleic acids carried internally to the recipient cell to participate in intracellular signaling. For example, exosomal miR-21 of renal tubular epithelial cell origin activates renal fibroblasts and promotes renal fibrosis by inhibiting the phosphatase and tensin homologue (PTEN)/protein kinase B (AKT) signaling pathway.^62^ The delivery of syndecan-1 attenuates ALI via the FAK/p190RhoGAP/RhoA/ROCK/nuclear factor kappa B (NF-κB) signaling pathway.^63^ Second, it binds to the receptor cells through membrane surface proteins and mediates the intracellular signaling cascade response in the receptor cells. For example, DC-derived exosomes can combine with bacterial toll-like receptor ligands to indirectly induce innate immune responses by enhancing the stimulation of bystander DCs.^64,65^ Third, when exosomes are internalized by recipient cells, they promote the production of new exosome populations by recipient cells.^66^ In addition to their primary role as carriers mediating biological effects within the target cells, exosomes play an indispensable role in liquid biopsies,^27^ cell-free vaccine development,^67^ drug delivery,^68^ and even regenerative medicine.^69^

## 3 Exosomal miRNAs

MicroRNAs are a class of noncoding single-stranded RNA molecules of approximately 22 nucleotides in length encoded by endogenous genes and are highly conserved in plants and animals. They can bind to the 3′ untranslated region or open reading frame of downstream target genes to modulate their expression at the posttranscriptional level and usually act as an inhibitor.^70^ The biogenesis of miRNAs is well understood by researchers.^71,72^ In animals, miRNA synthesis requires RNA polymerase II and two types of RNase III proteins (Drosha and Dicer). First, the gene carrying miRNA information is transcribed into pri-miRNA by RNA polymerase II in the nucleus. Second, pri-miRNA is cleaved in the nucleus by the Drosha enzyme into precursor miRNA (pre-miRNA) with an approximately 70 nucleotide length. Then, the pre-miRNA is transferred from the nucleus to the cytoplasm with the help of the exportin-5. In the cytoplasm, the pre-miRNA is shed from the exportin-5 and cleaved by the Dicer enzyme into a mature double-stranded miRNA (mature miRNA) of approximately 20 nucleotides in length.^73,74^ The mature miRNA eventually forms the RNA-induced silencing complex (RISC) with the Argonaute protein.^72^ The RISC is a ribonucleoprotein complex that guides miRNA to the target mRNAs to achieve gene silencing.^75^ In this process, the seven miRNA 5′-end nucleotides are the key to mRNA recognition.^76^ The miRNA-mediated gene-silencing modalities are mRNA degradation and mRNA translation inhibition. When the miRNA is precisely paired with the target mRNA base, the target mRNA is degraded. However, translational repression will occur when miRNAs are imperfectly paired with target miRNAs. miRNA degradation is irreversible, while mRNA translational repression is reversible because stable mRNAs can be translated again after eliminating translational repression.^77^ Exosomal miRNAs were found in human serum.^78^ Based on the peculiarity of miRNAs and the high abundance of miRNAs in exosomes, exosomal miRNAs have been recently shown to regulate various signaling pathways and phenotypes, serving as key regulators in many cancer, inflammatory, and metabolic diseases.^79−81^ In addition, exosomes can be stably preserved under different conditions, and miRNAs can be stably expressed in exosomes.^82−84^ Therefore, exosomal miRNAs still have some potential value in disease diagnosis.

## 4 Role of Exosomal miRNAs in the Pathogenesis of ALI

**Figure 2:**
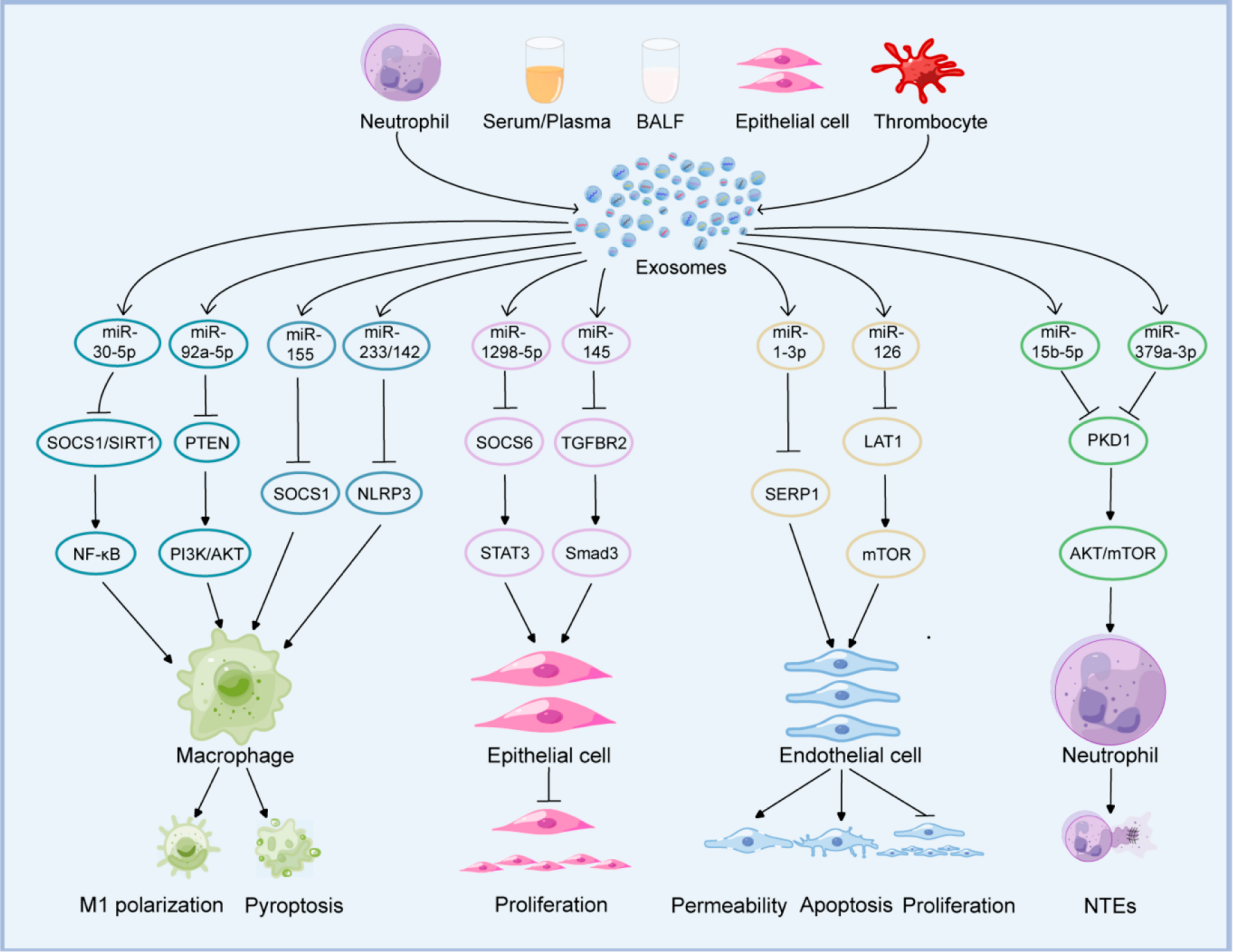
Pathogenesis of ALI regulated by exosomal miRNAs. Examples of exosomal miRNAs that play a role in ALI. Exosomes from different sources are involved in ALI by transferring specific miRNAs to regulate relevant genes and pathways, causing changes in target cell phenotype and function.

Lung homeostasis is the cornerstone of lung health and depends substantially on the pulmonary microenvironment. Communication among the pulmonary epithelium, endotheliocytes, and immune cells dominantly contributes to maintaining the balance of the pulmonary microenvironment.^85,86^ The dysfunctions of these cells can alter the pulmonary microenvironment, leading to lung inflammation and cancer development.^87−90^ Various resident cells from the lung can achieve intercellular communication by secreting exosomes carrying a specific high or low expression of miRNA, providing a novel theoretical foundation for the pathogenesis of ALI (Figure 2).

### 4.1 Exosomal miRNAs and Macrophages

In the human body, macrophages are a type of immune cell that prevents pathogen invasion and preserves physiological homeostasis. According to the different microenvironments, macrophages can be distinguished into M1 and M2 polarization states. M1-type macrophages can promote inflammatory responses by producing interleukin (IL)-6, TNF-α, IL-12, and other proinflammatory mediators. In contrast, M2-type macrophages have the capacity for anti-inflammatory responses and tissue repair. They produce IL-10, TGF-β, and other anti-inflammatory mediators to inhibit inflammatory responses and accelerate wound healing and revascularization.^91^ During ALI, exosomes from multiple cellular origins can deliver miRNAs to lung macrophages, causing macrophage activation and generation of inflammatory mediators to hasten lung inflammation. Studies have shown that TNF-α stimulation of neutrophils generates exosomes with a high miR-30d-5p expression that can reach the lungs of mice and induce NF-κB activation by targeting lung macrophage suppressors of cytokine signaling (SOCS) 1/sirtuin 1 (SIRT1), thereby resulting in M1 macrophage polarization and pyroptosis.^53^ In the lipopolysaccharide (LPS)-induced rat sepsis model, exosomes isolated from bronchoalveolar lavage fluid (BALF) highly expressed miR-92a-3p, which activates NF-κB by targeting the PTEN/AKT signaling pathways in alveolar macrophages, increasing inflammation and alveolar permeability in the rat lung.^92^ miR-155 is a common regulator of inflammation.^93^ In a different study of sepsis-induced ALI, elevated serum exosomal miR-155 caused macrophage proliferation and release of proinflammatory mediators by targeting lung macrophage SH2-containing inositol 5′-phosphatase 1 (SHIP1)/SOCS1, respectively. Inhibiting miR-155 may counteract the proinflammatory effects of macrophages.^21^ However, it has also been reported that miR-155 can alleviate the disease by reducing the formation of neutrophil extracellular traps (NETs) in the lungs of mice with abdominal sepsis through upregulation of peptidyl arginine deiminase 4 (PAD4) and promotion of histone 3 citrullination.^94^ In addition to increased levels of proinflammatory miRNAs in exosomes, anti-inflammatory miRNA levels are concurrently declining. Zhang et al.^95^ proved that exosomes isolated from BALF in mice with pneumonia were macrophage-derived. Moreover, the decrease of anti-inflammatory miR-223/142 in exosomes promoted the NOD-like receptor thermal protein domain associated protein 3 (NLRP3) inflammasome activation in macrophages, which subsequently caused the delivery of proinflammatory mediators (IL-1β and IL-18) to induce ALI. Moreover, a recent study showed that mitochondrial autophagy induced miR-138–5p promoter demethylation and inhibited NLRP3 inflammatory vesicle activation and macrophage pyroptosis, thereby attenuating septic lung injury.^96^

### 4.2 Exosomal miRNAs and Epithelial Cells

The alveolar epithelium is an important defense mechanism of the lung against external invasion. It generously covers the alveolar surface and functions as a lung protector, assisting in preserving the structural integrity of the lung during ALI.^97^ Two types of alveolar epithelial cells (AECs) are resident in the alveolar epithelium, namely, alveolar epithelial type I cells (ACE I) and alveolar epithelial type II cells (ACE II). ACE I, which covers approximately 95%–98% of the alveolar surface area, is majorly involved in the air–blood exchange between the alveoli and the blood. In contrast, ACE II, which occupies approximately 2%–5% of the surface area, can secrete surface-active substances to maintain alveolar surface tension.^98,99^ Exosomal miRNAs have been implicated in numerous studies as mediating the involvement of AECs in the emergence of ALI. SOCS6 acts as a member of the negative feedback regulation, reducing cytokine signaling by inhibiting multiple activated cytokines and tyrosine kinase receptors.^100^ Ma et al. cocultured serum exosomes from patients with septic lung injury with BEAS-2B cells and observed that exosomal miR-1298-5p could activate the downstream signal transducer and activator of the transcription 3 (STAT3) signaling pathway by suppressing SOCS6 expression in cells, resulting in the suppression of cell proliferation and an increase of cell permeability. Moreover, overexpression of SOCS6 could alleviate cell damage.^101^ miR-145 is thought to be a tumor suppressor.^102^ A study has revealed that miR-145 was significantly downregulated in blood exosomes from septic patients with lung injury and in LPS-treated BEAS-2B cells. Additionally, there was a positive correlation between the degree of miR-145 reduction and the condition of lung injury. Mechanistic studies indicate that miR-145 is significantly reduced in BEAS-2B cells by targeting TGF-β receptors 2, elevating the downstream Smad3, and promoting the inflammatory cytokines’ IL-2 and TNF-α deliverance to cause lung injury.^103^

### 4.3 Exosomal miRNAs and Endothelial Cells

Vascular endothelial cells participate in vascular tension formation, local blood flow regulation, immune response, and angiogenesis.^104^ Vascular endothelial damage due to disruption of the vascular endothelial barrier is a pathological characteristic of ALI.^105^ miR-1–3p expression drastically increased in the plasma exosomes of rats with septic-associated lung injury and LPS-stimulated human umbilical vein endothelial cells (HUVECs), which promoted apoptosis and cytoskeleton contraction and increased monolayer endothelial cell permeability by inhibiting stress-associated endoplasmic reticulum protein 1 (SERP1) expression in HUVECs.^106^ In 2019, severe acute respiratory syndrome coronavirus 2 (SARS-CoV-2) broke out, with a significant negative impact on the economy and global public health. Hypoxic respiratory failure caused by ALI is the leading cause of mortality in patients infected with SARS-CoV-2.^107^ Increased plasma abundance of exosome-associated neutrophil elastase was related to endothelial cell injury in patients with SARS-CoV-2 and ARDS.^108^ In addition, the elevation of serum exosomal miR-126 during severe community-acquired pneumonia (SCAP) may be associated with inhibiting pulmonary vascular endothelial cell proliferation and promoting apoptosis by targeting the L-type amino acid transporter 1 (LAT1)/mammalian target of rapamycin (mTOR) signaling axis.^109,110^

### 4.4 Exosomal miRNAs and Neutrophils

Neutrophils are first-line defense cells that eliminate pathogenic microorganisms through a nonspecific mechanism. The release of NETs, a unique mechanism of the natural immune response, is an important mode of action to locate and kill pathogens.^111^ However, NETs play a dual role. In the early stages of sepsis, NETs appear to have a protective function, but as the disease progresses, an enhanced NET release may contribute to thrombosis and multiorgan dysfunction.^112^ During infectious shock, miR-15b-5p and miR-378a-3p can advance the formation of NETs and aggravate lung injury by targeting neutrophil polycystin 1 (PKD1) and activating the AKT/mTOR autophagy pathway. In addition, the research team revealed for the first time that IκB kinase inhibitors can ameliorate the severity of lung injury during infectious shock by controlling the secretion of platelet-derived exosomes and inhibiting the formation of NETs.^113^

## 5 Role of Exosomal miRNAs in ALI Treatment

Despite the multiple targets for ALI/ARDS that have been researched, efficient clinical treatment for ALI is still absent. The main treatment for ALI/ARDS remains symptomatic, including mechanical ventilation, fluid management, corticosteroid supplementation, and inhaled pulmonary vasodilators.^14^ Although these treatments can relieve the symptoms of patients, they do not solve the underlying problem. Recently, RNA-mediated gene-silencing therapy has opened up a new path for treating ALI/ARDS.^114^ However, because of the characteristics of naked siRNA, the low efficiency of siRNA delivery to target cells limits the therapeutic performance of siRNA. Therefore, a carrier with good biocompatibility and transport capabilities is urgently needed.^115^ Exosomes offer excellent potential as carriers for gene therapy and drug delivery because of their unique physicochemical characteristics (small size, high penetration, high deliverability, and minimal immunogenicity).^116^ For example, exosome-loaded adriamycin exhibits a faster cellular uptake and more severe toxic side effects than free adriamycin and liposome-encapsulated adriamycin.^117^ Although miRNAs are relatively poor matches to target mRNAs compared to siRNAs, they also have gene-silencing effects.^75^ Evidence suggests that mesenchymal stem cell (MSC)-derived exosomal miRNAs can form intercellular interactions with resident lung cells and have potential therapeutic value for ALI by targeting different genes and pathways (Table 1).

**Table 1:** Pathogenesis of Exosomal miRNAs in the Ttreatment of ALI

Source	miRNA	Model construction	Effector cells	Mechanism	Function	Reference
BMSCs	miR-199a-3p	LPS	Primary mice ATIIC	Increasing of α/γ-ENaC	Inhibited apoptosis, increased cell viability, and restored pulmonary edema	(120)
BMSCs	miR-30b-3p	LPS	MLE-12	Targeting SAA3	Inhibited apoptosis and promoted cell proliferation	(121)
BMSCs	miR-132-3p	LPS	MLE-12	Targeting PTEN/PI3K/AKT signaling pathway	Promoted cell proliferation and inhibited apoptosis	(122)
BMSCs	miR-182-5p, miR-23-3p	LPS	MLE-12	Targeting Ikbkb/Usp50 and inhibiting NF-κB/Hedgehog signaling pathway	Reduced EMT generation	(124)
BMSCs	miR-150	LPS	HUVEC	Targeting MAPK signaling pathway	Inhibited apoptosis	(125)
BMSCs	miR-384-5p	LPS	NR8383	Targeting Beclin-1	Inhibited apoptosis and cellular autophagic stress	(126)
BMSCs	miR-127-5p	LPS	293T	Targeting CD64	Reduced NET formation	(127)
BMSCs	miR-181-5p	LPS	MDM	Targeting PTEN/pSTAT1/SOCS1 signaling pathway	Promoted macrophage reprogramming	(129)
BMSCs	miR-425	High oxygen	RL6-6TN	Targeting PTEN/PI3K/AKT signaling pathway	Increased cell viability and inhibited apoptosis	(130)
BMSCs	miR-21-5p	I/R	Primary lung endothelial cells	Targeting PTEN/PDCD4	Inhibited apoptosis	(131)
BMSCs	miR-202-5p	I/R	MLE-12	Targeting CMPK2	Inhibited pyroptosis	(132)
UC-MSCs	miR-22-3p	LPS	NR8383	Targeting FZD6 to inhibit NF-κB activation	Inhibited inflammation	(134)
UC-MSCs	miR-377-3p	LPS	HPAEpiC	Targeting RPTOR	Promoted autophagy	(135)
UC-MSCs	miR-199a-5p	SM	BEAS-2B	Targeting CAV1/NRF2 signaling pathway	Inhibited oxidative stress	(136)
UC-MSCs	miR-451	Burns	–	Targeting TLR4 to inhibit NF-κB	Inhibited inflammation	(137)
UC-MSCs	miR-451	Burns	NR8383	Targeting MIF to activate the PI3K/AKT signaling pathway	Promoted macrophage transformation from M1 to M2	(138)
UC-MSCs	miR-146-5p	Pristane	Primary lung macrophages	Inhibiting NOTCH	Promoted macrophage transform from M1 to M2	(139)
EPCs	miR-126	CLP	HUVEC	Targeting SPRED1 to activate the RAF/ERK signaling pathway	Increased endothelial cell permeability, promoted endothelial cell proliferation, migration, and revascularization	(141)
EPCs	miR-126-3p, miR-126-5p	LPS	SAEC	Targeting PIK3R2 to inhibit HMGB1/VEGF	Maintenance of alveolar epithelial barrier integrity	(142)
EPCs	miR-382-3p	CLP	–	Targeting BTRC/IκBα/NF-κB	Mediated tissue repair and T-cell immune activity	(143)
ADSCs	miR-125b-5p	LPS	PMVEC	Targeting Keap1/Nrf2/GPX4 signaling pathway	Inhibited ferroptosis	(146)
ADSCs	miR-126	Histone	HUVEC	Targeting PI3K/AKT signaling pathway	Inhibited apoptosis	(150)
BALF	miR-223-3p	LPS	NR8383	Targeting STK39	Increased cell viability, activate autophagy, reduced apoptosis, and inflammation	(151)
BEAS-2B	miR-103a-3p	LPS	BEAS-2B	Targeting TBL1XR1 to inhibit NF-κB	Inhibited inflammation	(152)
RLE-6TN	miR-146a	LPS	NR8383	Targeting TLR4 to inhibit NF-κB	Inhibited inflammation	(153)
bEnd.3	miR-125-5p	LPS	–	Targeting TOP2A to elevate VEGF	Inhibited apoptosis	(154)
A549	miR-371b-5p	Bleomycin	ATIIC	Targeting PTEN to promote AKT/GSK3β/FOXO phosphorylation	Promoted cell-specific proliferation	(155)

### 5.1 BMSC-Derived Exosomal miRNAs and ALI

Bone marrow mesenchymal stem cells (BMSCs) are a type of adult stem cell in the bone marrow other than hematopoietic stem cells. They are used to treat ALI because of their immunomodulatory and regenerative properties, which can lessen the generation of proinflammatory cytokines and promote tissue repair.^118^ BMSC-derived exosomes (BMSCs-Exo) may contribute to their capacity to cure ALI.^119^ BMSCs-Exo were reported to reduce LPS-induced ALI by promoting the viability of mouse type II AECs (ATIIC) while inhibiting their apoptosis. Elevated miR-199a-3p in exosomes causes an increase in the α/γ-epithelial sodium channel protein, which helps to restore pulmonary edema.^120^ By coculturing BMSCs-Exo with the mouse lung epithelial cell line (MLE-12), elevated miR-30b-3p/miR-132-3p in exosomes could target and inhibit the serum amyloid A isoform 3 (SAA3)/TNF receptor associated factor 6 (TRAF6) expression in MLE-12 cells, thereby inhibiting apoptosis and promoting cell proliferation to improve LPS-induced ALI.^121,122^ Epithelial–mesenchymal transition (EMT) is the conversion of epithelial cells to mesenchymal cells, closely related to the occurrence and progression of idiopathic fibrosis.^123^ Xiao et al.^124^ have shown that BMSCs-Exo could inhibit the expression of nuclear factor kappa B kinase subunit beta (IKBKB) and ubiquitin-specific peptidase 50 (Usp50) in MLE-12 cells by delivering miR-182-5p and miR-23-3p, respectively. As binding of IKBKB to Usp50 can cause I kappa B kinase beta (Ikkβ) ubiquitination, inhibition of IKBKB and Usp50 caused a reduction in Ikkβ ubiquitination, which in turn blocked NF-κB and hedgehog signaling pathway activation, reversing EMT progression. In the LPS-induced pulmonary microvascular endothelial cell model, the BMSC-derived exosomal miR-150 can degrade the apoptosis of pulmonary microvascular endothelial cells by inhibiting the mitogen-activated protein kinase (MAPK) signaling pathway activation to maintain the structural integrity of alveoli.^125^ Furthermore, BMSC-derived exosomal miR-384-5p can reduce macrophage apoptosis and autophagy stress by targeting Beclin-1 of alveolar macrophages to improve survival in ALI rats.^126^ In animal model studies, BMSC-derived exosomal miR-127-5p inhibited the formation of NETs in sepsis-associated ALI by targeting CD64.^127^ Macrophage reprogramming has a protective effect on inflammation.^128^ It was reported that miR-181a-5p in BMSC-derived EVs downregulated PTEN, subsequently producing pSTAT1 and SOCS1. Activating this signaling axis promoted macrophage reprogramming, reduced secretion of TNF-α and IL-8, and inhibited the inflammatory response in ARDS.^129^

In addition to LPS-induced ALI, BMSCs-Exo has been studied in hyperoxia-induced ALI. High expression of miR-425 in MSCs-Exo can target and inhibit the expression of PTEN in rat alveolar epithelial type II cell lines. PTEN is an antioncogene, and when it is inhibited, the downstream phosphoinositide 3-kinase (PI3K)/AKT inflammatory signaling pathway is activated, resulting in increased cell activity and decreased apoptosis, thus alleviating cell damage.^130^ Lung ischemia/reperfusion (I/R) is also a cause of ALI. Ji et al. found that BMSCs-Exo had protective effects against oxidative stress-induced ALI in mice by constructing a mouse I/R model *in vivo* and a hypoxia/reoxygenation model *in vitro*, which was attributed to the inhibition of endogenous and exogenous apoptosis by exosomal miR-21-5p by inhibiting the PTEN and programmed cell death 4 (PDCD4) in primary mouse pulmonary endothelial cells.^131^ In another study of a mouse model of I/R, miR-202-5p from BMSCs-Exo could inhibit pyroptosis in lung epithelial cells by targeting cytidine/uridine monophosphate kinase 2 (CMPK2).^132^

### 5.2 Umbilical-Cord-Blood MSC-Derived Exosomal miRNAs and ALI

Umbilical-cord-blood MSCs (UCB-MSCs) are typical adult stem cells. They are considered the optimum selection for stem cell therapy because of their noninvasive collection, low immunogenicity, easy *in vitro* expansion, and ethical compliance compared with other stem cells.^133^ Numerous studies have pointed to the ability of UCB-MSC-derived exosomes (UCBMSCs-Exo) to mitigate the progression of ALI. Zheng et al.^134^ found in the LPS-induced macrophage model that the elevated miR-22-3p in UCBMSCs-Exo could target and inhibit frizzled class receptor 6 (FZD6), reduce the cellular inflammatory response and oxidative stress, promote cell proliferation, and inhibit cell apoptosis. In addition, animal experiments have shown that exosomal miR-22-3p can reduce lung inflammation by inhibiting NF-κB activation. Autophagy plays an essential role in tissue repair. Lung inflammation and oxidative stress can be significantly inhibited in pulmonary diseases by activating autophagy. Wei et al.^135^ found that miR-377-3p in UCBMSCs-Exo could target and inhibit the regulatory-associated protein of mTOR complex 1 (PRTOR) in human AECs and promote cellular autophagy to protect against LPS-induced ALI. Moreover, miR-199a-5p from UCBMSCs-Exo was shown to be a key molecule to alleviate sulfur mustard (SM)-related oxidative stress by regulating the caveolin 1 (CAV1)/nuclear factor erythroid-2–related factor 2 (NRF2) signaling pathway.^136^ Burns are also a cause of ALI, and burn-induced ALI requires the involvement of functional toll-like receptor 4 (TLR4).^8^ It was discovered that elevated miR-451 in UCBMSCs-Exo could target and inhibit TLR4 in mouse lung tissue, thereby blocking NF-κB activation and reducing proinflammatory cytokine generation to alleviate burn-induced ALI.^137^ However, the specific mechanism of action of miR-451 still needs further clarification in *in vitro* experiments. In another study of burn-induced ALI, the mechanism of the effect of miR-451 in HUC-MSCs-Exo was confirmed at the cellular level, which promoted the transformation of macrophages from M1 to M2 by targeting the macrophage migration inhibitory factor (MIF)/PI3K/AKT signaling pathway.^138^ Relevant reports have found that UCBMSCs-Exo also has a therapeutic effect on systemic lupus erythematosus (SLE)-associated diffuse alveolar hemorrhage in mice. The exosomes reduced the level of NOTCH expression in diffuse alveolar hemorrhage (DAH) mice via lung tissue via miR-146a-5p, thus reducing lung tissue bleeding and inflammatory cell infiltration. This may be due to miR-146a-5p facilitating the transformation of alveolar macrophages from M1 to M2.^139^

### 5.3 Endothelial-Progenitor-Cell-Derived Exosomal miRNAs and ALI

Endothelial progenitor cells (EPCs) are a special type of stem cells and vascular endothelial precursor cells, with a role in normal endothelial function and repair after vascular injury.^140^ In the ALI, EPCs can migrate to the site of the lesion and improve lung inflammation by participating in vascular endothelial remodeling. Many studies have revealed that miRNAs from EPC-derived exosomes (EPCs-Exo) influence this process. miR-126 is an important regulator of angiogenic signaling that maintains the integrity of vascular endothelial cells and the vascular detail. Xu et al.^141^ found that miR-126 in EPCs-Exo was transferred to HUVECs and activated the RAF/extracellular regulated protein kinase (ERK) signaling pathway by targeting the sprouty-related EVH1 domain containing 1 (SPRED1) in cells, increasing endothelial cell permeability while promoting endothelial cell proliferation, migration, and angiogenesis to alleviate ALI. Another study found that intratracheal dripping of EPC-Exo with high expression of miR-126-3p and miR-126-5p reduced lung edema and inflammatory cell infiltration and restored alveolar epithelial barrier integrity in mice after 24 and 48 h of LPS induction. This may be connected to suppressing the ALI-related target genes phosphoinositide-3-kinase regulatory subunit 2 (PIK3R2) and high mobility group box 1 (HMGB1)/vascular endothelial growth factor (VEGF) by miR-126-3p/miR-126-5p.^142^ In addition, miR-382-3p in EPCs-Exo can also target the regulation of a beta-transducin repeat containing E3 ubiquitin protein ligase (BTRC) and an IκBα/NF-κB axis to restore the number of lymphocytes and maintain the balance between Th1 and Th2 cells to alleviate ALI in mice with cecal ligation and puncture (CLP)-induced sepsis.^143^

### 5.4 Adipose-Stem-Cell-Derived Exosomal miRNAs and ALI

Adipose stem cells (ADSCs) are mesenchymal ASCs with a multifunctional differentiation potential isolated from adipose tissue. It is of great significance in treating diseases because of its ability to repair tissue cells, resist aging, and improve the subhealth state of the body.^144^ For instance, human ADSC-derived exosomes (ADSCs-Exo) could inhibit oxidative stress in pulmonary vascular endothelial cells and reduce cell monolayer permeability to alleviate CLP-induced ALI in mice.^145^ Moreover, miR-125b-5p in the ADSCs-Exo could alleviate ferroptosis of pulmonary vascular endothelial cells (PMVECs) in sepsis-associated ALI by regulating the Kelch-like ECH-associated protein 1 (Keap1)/nuclear factor-erythroid 2-related factor 2 (Nrf2)/glutathione peroxidase 4 (GPX4) signaling axis, thereby reducing lung inflammation.^146^ There is growing evidence that extracellularly released histones are becoming damage-associated molecular patterns, which are thought to contribute to the development of lung injury.^126,147−149^ Mituzn et al.^150^ showed that miR-126 in ADSCs-Exo inhibited PI3K/AKT activation in endothelial cells and attenuated histidine-induced ALI.

### 5.5 Others

In addition to stem cell therapy, the communication of exosomal miRNAs produced by lung resident cells is also a potential therapeutic modality for ALI. It has been suggested that AEC-derived exosomes may also alleviate ALI by acting on macrophages. Nan et al.^151^ found that BALF-Exo from LPS-induced ALI mice could wrap miR-223-3p to reach alveolar macrophages and negatively regulate serine/threonine kinase 39 (STK39) in the cells. This boosted cell survival, activated autophagy, and reduced apoptosis and inflammation, alleviating ALI. Li et al.^152^ found that miR-103a-3p was lowly expressed in exosomes generated from serum samples of pediatric patients with pneumonia and the LPS-induced human lung epithelial cell line (BEAS-2B). miR-103a-3p could target transducin of (beta)-like 1 X-linked receptor 1 (TBL1XR1) in BEAS-2B to inhibit NF-κB activation, releasing proinflammatory mediators IL-6 and TNF-α. Traditional Chinese medicine (TCM) is a national treasure of China, and salidroside is one of the effective monomeric components of the TCM *Rhodiola rosea* L. Zheng et al.^153^ have shown that salidroside treatment elevated miR-146a in alveolar epithelial-derived exosomes, which targeted and inhibited TLR4 in alveolar macrophages causing NF-κB activation to improve LPS-induced ALI in rats, providing a new theory for the treatment of ALI with TCM. However, exosomal miRNAs produced by cells are not limited to crosstalk with other cells to exert their functions but could also act on themselves. In a mouse model of sepsis constructed by CLP, miR-125b-5p was highly expressed by endothelial cell-derived exosomes, which could inhibit apoptotic injury by targeting and inhibiting their own DNA topoisomerase II alpha (TOP2A) and elevating the vascular endothelial growth factor (VEGF).^154^ In addition, the ATIIC-derived exosomal miR-371b-5p could promote AKT and its downstream glycogen synthase kinase-3 beta (GSK3β) and forkhead box O (FOXO) phosphorylation by targeting and inhibiting its own PTEN, thus causing ATIIC-specific proliferation and promoting damaged alveolar re-epithelialization to alleviate bleomycin-induced ALI.^155^

## 6 Role of Exosomal MiRNAs in the Diagnosis of ALI

Biomarkers are biochemical indicators that can objectively detect and evaluate changes in body structure and are used for disease screening, prediction, and diagnosis.^156^ However, there is no single biomarker with sufficient sensitivity and specificity for clinical diagnosis of ALI. Therefore, new biomarkers for the early detection of ALI are urgently required. In the past decade, miRNAs have been discovered to engage in pathophysiological processes, including lung injury and repair. Consequently, scientists have been actively investigating their potential for use as biomarkers.^157,158^ miRNAs have many advantages as biomarkers. First, the internal cargo of exosomes is finely regulated by parental cells in physiological and pathological states. This can reflect changes in parental cells to a certain extent.^159^ Therefore, the internal cargo of exosomes is likely to be a variable feature that can be captured to predict the functional state of the parent cells. Second, exosomes are relatively stable based on their characteristics, and exosome miRNAs are more resistant to degradation after cryopreservation than cellular miRNAs.^160^ Most importantly, exosomes are present in almost all biofluids, providing a good biological basis for exosomes as a disease diagnostic tool, especially for noninvasive diagnostics.^161^ In summary, exosomal miRNAs are superior to circulating or cellular miRNAs alone as diagnostic tools.

With the continuous advancement and application of various bioassay technologies, Sandfeld-Paulsen et al.^162^ identified various highly expressed exosomal proteins using mass spectrometry analysis of plasma exosome proteomics in patients with lung cancer. These proteins could be used as a diagnostic tool for lung cancer independently of pathological staging and histological subtypes. In addition, the authors also demonstrated that various exosomal miRNAs of humoral origin can be used as early diagnostic markers for ALI to some extent. Parzibut et al.^163^ compared the expression of plasma exosomal miRNA in 8 patients with ARDS and 10 healthy subjects using small RNA sequencing analysis and identified 12 differentially expressed miRNAs. Among these differentially expressed miRNAs, seven miRNAs (miR-221-3p, miR-24-3p, miR-130a-3p, Let-7d-3p, miR-1273a, miR-98-3p, and miR-193a-5p) were proved to distinguish ARDS and hemorrhagic shock well using receiver operating characteristic curve analysis (area under the curve >0.8). Recent studies have found that EVs with CD14^+^ in BALF can also serve as a new biomarker for ARDS. Elevated counts of EVs with CD14^+^/CD81^+^ in BALF of patients with sepsis-associated ARDS are associated with the increased mortality of patients with ARDS.^164^ SCAP usually leads to high mortality in ARDS. Higher levels of miR-146a, miR-126, miR-27a, and miR-155 were found in serum exosomes of patients with SCAP than in the non-ARDS group, and the combination of the four was predictive of ARDS. In addition, the authors also found that miR-126 could predict 28-day mortality in patients with SCAP.^109^ In a recent asthma study, miR-126 was highly expressed in serum exosomes of patients with allergic asthma and lung tissues of asthmatic mice, which has some reference value for diagnosing bronchial asthma.^165^ Moreover, the amount of miR-122-5p in plasma EVs was proportional to the number of inflammatory cells in the blood of patients with asthma, and it has the potential to distinguish different subtypes of asthma.^166^ Sputum is also the direct body fluid for respiratory disease detection. miR-142-3p, miR-223-3p, and miR-629-3p were increased in the sputum of patients with severe asthma, and this was associated with increased sputum neutrophils.^167^ It would be interesting to prove that these miRNAs are enriched in exosomes. Harmful gas inhalation is an etiology of ALI. In ozone-induced ALI mice, differential miRNAs in EVs isolated from BALF increased with increasing ozone concentration compared with controls, among which miR-22-3p is expected to be a marker of ALI. In addition, comprehensive analysis suggests that the occurrence of ALI induced by miR-22-3p could be by targeting macrophages.^168^ Local vascular inflammation due to intimal tear and pseudotumor formation in acute type A aortic dissection (ATAAD) can progress to systemic inflammatory syndrome, leading to ALI. miR-485 was upregulated, and miR-206 was downregulated in plasma exosomes of patients with ALI compared with those with ATAAD without ALI and is expected to be a marker of ALI in patients with ATAAD.^169^

## 7 Opportunities and Challenges for Exosomal miRNA in ALI

In previous studies of ALI, exosomes seem to be a new therapeutic agent for gene and drug delivery. They are also helpful in early diagnosis and improve the prognosis (Table 2). However, there are still many challenges to using exosomes for practical clinical applications: (1) The mechanism of action of exosomal miRNAs is mostly based on animal/cellular models; it needs more validation in clinical samples. (2) Prospective studies of clinical samples are mostly based on small samples and lack validation in large cohort studies. (3) The regulation mechanism of miRNA in ALI is complex and affected by many factors. Further elucidation of the mechanism of action of exosomal miRNAs in disease states is needed. (4) The isolation of exosomes is uneven and relatively costly, which undoubtedly increases the cost of treating patients if applied to clinical treatment. Therefore, to apply exosome therapy to clinical practice, we must continue exploring exosome isolation technology to find an efficient, rapid, low-cost, and standardized extraction method. (5) EVs include several subtypes, and different EVs may have different effects; therefore, there is an urgent need for specific ways to accurately distinguish different subtypes of EVs and ensure the purity of exosomes. (6) Although exosomes as a diagnostic tool in liquid biopsies are easy to obtain and noninvasive, the changes of miRNA in exosomes in diseased states are diverse and even change continuously with the development of ALI. Combining multiple exosomal miRNAs with existing mature diagnostic indicators may help in the early diagnosis of ALI and improve the sensitivity and specificity of ALI diagnosis. (7) Differences in exosomes secreted by different cells under different environments/stimuli and how to prepare stable decoy exosomes for disease treatment must also be addressed.

**Table 2:** Exosomal miRNAs Associated with ALI Diagnosis

Diseases	Biofluids	miRNAs	Changes	Reference
ARDS	Plasma	miR-130a-3p, miR-98-3p, miR-221-3p, miR-193a-5p, miR-24-3p, Let-7d-3p, miR-1273a, miR-193a-5p	Upregulated	(163)
SCAP	Serum	miR-146a, miR-126, miR-27a, miR-155	Upregulated	(109)
	Serum/lung tissue	miR-126	Upregulated	(165)
ASTHMA	Plasma	miR-122-5p	Upregulated	(166)
	Sputum	miR-142-3p, miR-223-3p, miR-629-3p	Upregulated	(167)
ALI	BALF	miR-22-3p	Upregulated	(168)
ATAAD	Serum	miR-485, miR-206	Upregulated, Downregulated	(169)

## 8 Conclusion

In conclusion, exosomal miRNAs have a multifaceted regulatory role in ALI. As a new class of regulators, miRNAs reach effector cells under exosomal load to mediate intercellular crosstalk. These miRNAs activate the inflammatory signaling axis by regulating downstream target genes, promoting changes in the phenotype and function of target cells to promote disease progression. Moreover, MSCs are a class of pluripotents with self-renewal and multidirectional differentiation capabilities. The exosomes produced by MSCs from different sources achieve anti-inflammatory, antiapoptotic, and antioxidative stress effects through miRNAs, which are expected to be applied in clinical cell-free therapy. In addition, exosome substances are protected from degradation and may be useful in diagnosing ALI by analyzing the composition of miRNAs with specificity in humoral exosomes. Although the research based on miRNA delivery is only the tip of the iceberg, with the continuous progress of technology and the gradual deepening of research, exosomal miRNAs are expected to provide a new strategy for preventing, diagnosing, and treating ALI.

## Abbreviations Used

ALI: acute lung injury
ARDS: acute respiratory distress syndrome
PaO_2_: arterial pressure of oxygen
FiO_2_: inspiratory fraction of oxygen
EVs: extracellular vesicles
ISEV: International Society for Extracellular Vesicles
MISEV: Minimum Information for Studies of Extracellular Vesicles
MVBs: multivesicular bodies
ESCRT: endosomal sorting complexes required for transport
PIP2: phosphatidylinositol 4,5-bisphosphate
SEM: scanning electron microscope
TEM: transmission electron microscopy
DCs: dendritic cells
MHC I: major histocompatibility complex I
MHC II: major histocompatibility complex II
Hsp: heat shock protein
Alix: ALG-2-interacting protein X
TSG101: tumor susceptibility gene 101
TGF-β: tumor growth factor-beta
TNF-α: tumor necrosis factor-alpha
TRAIL: TNF-related apoptosis-inducing ligand
GAPDH: glyceraldehyde 3-phosphate dehydrogenase
PKM2: pyruvate kinase M2
PTEN: phosphatase and tensin homologue
AKT: protein kinase B
NF-κB: nuclear factor kappa B
pre-miRNA: precursor miRNA
mature miRNA: mature double-stranded miRNA
RISC: RNA-induced silencing complex
IL: interleukin
SOCS1: suppressor of cytokine signaling
SIRT1: sirtuin 1
LPS: lipopolysaccharide
BALF: bronchoalveolar lavage fluid
SHIP1: SH2-containing inositol 5′-phosphatase 1
NETs: neutrophil extracellular traps
PAD4: peptidyl arginine deiminase 4
NLRP3: NLR family pyrin domain containing 3
AECs: alveolar epithelial cells
ACE I: epithelial type I cells
ACE II: alveolar epithelial type II cells
STAT3: signal transducer and activator of transcription 3
HUVECs: human umbilical vein endothelial cells
SERP1: stress-associated endoplasmic reticulum protein 1
SARS-CoV-2: severe acute respiratory syndrome coronavirus 2
SCAP: severe community-acquired pneumonia
LAT1: L-type amino acid transporter 1
mTOR: mammalian target of rapamycin
PKD1: polycystin 1
MSCs: mesenchymal stem cells
BMSCs: bone marrow mesenchymal stem cells
BMSCs-Exo: BMSC-derived exosomes
ATIIC: type II AECs
SAA3: serum amyloid A isoform 3
TRAF6: TNF receptor associated factor 6
EMT: epithelial–mesenchymal transition
IKBKB: inhibition of nuclear factor kappa B kinase subunit beta
Usp50: ubiquitin-specific peptidase 50
Ikkβ: IkappaB kinase beta
MAPK: mitogen-activated protein kinase
PI3K: phosphoinositide 3-kinase
I/R: ischemia/reperfusion
PDCD4: programmed cell death 4
CMPK2: cytidine/uridine monophosphate kinase 2
UCB-MSCs: umbilical cord blood mesenchymal stem cells
UCBMSCs-Exo: UCB-MSC-derived exosomes
FZD6: frizzled class receptor 6
PRTOR: regulatory-associated protein of mTOR complex 1
SM: sulfur mustard
CAV1: caveolin 1
NRF2: nuclear factor erythroid-2-related factor 2
TLR4: toll-like receptor 4
MIF: migration inhibitory factor
SLE: systemic lupus erythematosus
DAH: diffuse alveolar hemorrhage
EPCs: endothelial progenitor cells
EPCs-Exo: endothelial progenitor cell derived exosomes
ERK: extracellular regulated protein kinases
SPRED1: sprouty-related EVH1 domain containing 1
PIK3R2: phosphoinositide-3-kinase regulatory subunit 2
HMBG1: high mobility group box 1
VEGF: vascular endothelial growth factor
BTRC: beta-transducin repeat containing E3 ubiquitin protein ligase
CLP: cecal ligation and puncture
ADSCs: adipose stem cells
ADSCs-Exo: ADSC-derived exosomes
PMVECs: pulmonary vascular endothelial cells
Keap1: Kelch-like ECH-associated protein 1
Nrf2: nuclear factor–erythroid 2-related factor 2
GPX4: glutathione peroxidase 4
STK39: serine/threonine kinase 39
TBL1XR1: transducin (beta)-like 1 X-linked receptor 1
TCM: traditional Chinese medicine
TOP2A: DNA topoisomerase II alpha
VEGF: vascular endothelial growth factor
GSK3β: glycogen synthase kinase-3 beta
FOXO: forkhead box O
ATAAD: acute type A aortic dissection
